# To Solve the Coronavirus Crisis: Click Here

**DOI:** 10.1007/978-3-030-65355-2_18

**Published:** 2021-03-20

**Authors:** Esther Keymolen

**Affiliations:** 1 Tilburg University, Tilburg, The Netherlands; 2 Tilburg University, Tilburg, The Netherlands; 3 Tilburg University, Tilburg, The Netherlands; 4 Tilburg University, Tilburg, The Netherlands; Tilburg Institute of Law, Technology and Society, Tilburg Law School, Tilburg, The Netherlands

## Abstract

Human beings are technical beings. From the clothes we wear to the spaceships we fire into the sky, all these technologies are developed with the aim to protect ourselves, improve ourselves, and control the fickle world in which we live. Therefore, it should not come as a surprise that when hit with one of the biggest health crises of the last century, all over the world, governments have turned to technology to contain this life-threatening event. Most of these proposed—or already developed—technological solutions are data-driven.

Just as the turn to technology to solve this crisis does not come as a surprise, neither does the protest it has caused. Critical citizens and civil rights organizations worry about the possibility of personal data being shared with private parties, about governments ending up using the collected information against citizens, and they fear an overall loss of privacy and freedom if these applications became widely used. Overall, they suspect that what is introduced as a temporary instrument to counter this crisis will have long-lasting effects on society.

Human beings are technical beings. From the clothes we wear to the spaceships we fire into the sky, all these technologies are developed with the aim to protect ourselves, improve ourselves, and control the fickle world in which we live. Therefore, it should not come as a surprise that when hit with one of the biggest health crises of the last century, all over the world, governments have turned to technology to contain this life-threatening event. Most of these proposed—or already developed—technological solutions are data-driven: contact-tracing apps to establish with whom an infected person interacted, apps to provide proof that someone is not ill and can therefore access a building or public transport, facial recognition to identify people in the street or to check whether they are effectively quarantined, etc.

Just as the turn to technology to solve this crisis does not come as a surprise, neither does the protest it has caused. Critical citizens and civil rights organizations worry about the possibility of personal data being shared with private parties, about governments ending up using the collected information against citizens, and they fear an overall loss of privacy and freedom if these applications became widely used. Overall, they suspect that what is introduced as a temporary instrument to counter this crisis will have long-lasting effects on society.

In the Netherlands, the Prime Minister and the Minister of Health tried to calm things down by stating that they “did not suddenly pick up a new hobby and were definitely not interested in finding out individual citizens’ whereabouts.” Moreover, they assured that sufficient checks and balances would be implemented, applications developed and tested before the public eye in an “appaton,” and—naturally—the end result would be GDPR proof.

The underlying assumption of this line of defense is that the technology itself is not a matter of big concern as long as it is properly embedded in the social, technical, and economic context. Of course, we have to make sure that sufficient democratic safeguards are set in place, oversight is taken care of, and all legal requirements are abided by; then we would be fine. Or not?

In 1980, political philosopher Langdon Winner (1980) wrote a—now—famous and foundational book chapter and article called “Do artifacts have politics?” In this work, he argues that technologies are not just neutral objects being kept in check by the socio-technical context in which they function but that they are also politically significant in their own right. In a powerful manner, technologies can transform human aspirations, Winner claims. Therefore, they deserve our specific attention. It is not sufficient to only look at the context in which a technology is embedded or to count on good intentions, as the Dutch Prime Minister seems to suggest. We should also closely analyze the political qualities of the technology itself. In other words, we have to lay bare how technologies can steer the societal arrangements of power and control as well as how they impact the interactions that take place within these arrangements.

Whereas almost half a century ago, Winner predominantly focused on the political power of large energy systems such as nuclear plants, solar panels, and flood-control dams, I found his seminal work to still be extremely useful to grasp the political challenges we face when introducing data-driven solutions in times of crisis.

## Settling Societal Issues Through Technology

By and large, Winner distinguishes two ways in which technologies can have political qualities. The first way is rather straightforward. A technology can be used to settle a certain societal problem. For instance, the COVID-19 contact-tracing app is promoted as a way to quickly inform people who might be at risk of having caught the virus. However, this is not necessarily the only goal such an app could serve. One can also think of other uses, which are not communicated to the public but are, nevertheless, intentionally being built into the technology. It has been suggested that malicious companies could design a hidden backdoor into the app in order to collect and then monetize data. Also, theories of governments spying on their citizens or merely introducing the app to save on health costs have been voiced. These examples all presuppose a malicious actor who intentionally makes use of the design of a technology to pull the strings without people noticing. The technology, like a Trojan horse, disguises the political intervention taking place.

Some may think the idea of a malicious actor intentionally hijacking a coronavirus app is too far-fetched. For Winner, bad intentions are not a necessary condition for technologies to possess political properties. Actually, more often, technology has unintended political consequences. For instance, people might become overly confident because of the mere presence of the app and no longer strictly follow the social distancing rules. The app then ends up doing more harm than good. Or, in the long run, the app might be an enormous boost for e-health, leading care insurers to push for all kinds of new data-driven solutions, cutting back on financial compensation for face-to-face interactions. 50 years from now, historians might trace the start of their completely data-driven health care system back to the COVID-19 solutions that are now being introduced globally.

Whether or not you think this is a future worth wanting, it certainly illustrates that technologies can have far-reaching, political consequences without anyone intentionally introducing the technology with such goals in mind. Depending on one’s position in society, the power to influence the course of a technological innovation, to domesticate it, avoid it, or completely hack it will differ. Especially in the first phase, when the technology is still the most moldable, decisions will be made that will materialize and become fixed in the technology itself and in the practices that it will mediate, now and in the future. Keeping this technological perseverance in mind, we should not leave it to companies, individual data scientists, or politicians only to decide on the design and functionality of such impactful data-driven solutions.

## Inherently Political Technologies

Whereas the previous examples illustrate how the design of a technology—both intentionally and unintentionally—can influence and steer power and control in society, it also becomes clear that its political impact is not inherent to the technology as such. For instance, if we acknowledge that the digitalization of health might have negative effects on our overall health care system, we can adjust it.

Winner claims, however, that there is a second category of technologies that lacks this kind of flexibility and is actually inherently political. With that, he means that to choose for a certain technology is to also choose for a particular form of political life. In order to function properly, some technologies require a social setting to be organized in a very specific way. For instance, to choose for nuclear energy is to choose for a highly controlled and hierarchical organization as the security risks of such a technology are extremely high. Or, in the case of the contact-tracing app, to choose for an app is to choose for a public of smartphone owners. As a result, elderly people, poor people, and people who on ideological grounds refuse to have a smartphone might be overlooked and excluded, missing out on vital information or early diagnosis and treatment because all health systems become geared towards the functioning of the app.

It is also choosing for an infrastructure controlled by private companies. In order for the tracing app to function properly, it is claimed that we need to work with the decentralized Bluetooth infrastructure released by Apple and Google. At first sight, this might look like a mere technicality—if these companies enable an interoperable infrastructure, which will make the app functional; that would be great! However, it actually also entails an enormous power shift in favor of two of the most powerful tech companies in the world. For example, it would push citizens to choose for their devices if they wanted to make use of the app. It would also open the door for these companies to shape public health care policies and steer the political agenda (Sharon 2020). Even without breaching individual privacy, these companies would be able to gain valuable insights in the way communities go about their everyday lives (Veale 2020).

It is certainly not easy to detect the inherent political aspects of a technology. Indeed, because these infrastructural choices are framed as mere practical issues, in-depth democratic debate and control are lacking. In order words, to choose for this tracing app is to choose for accepting an infrastructure that is completely in the hands of two US-based tech giants who have interests and key values that do not necessarily align with those of a democratic society.

In times of crisis, it comes almost natural to policy-makers to turn to technology to help them govern a new and complex situation. However, technology is not a neutral instrument and can impose all kinds of values and restrictions on society. What we can learn from Winner is that, while we might think that we are developing technological tools to control this crisis, we are actually building the political life of our post-corona society: the political life of our new common.

## References

[CR1] Sharon T (2020) “When Google and Apple get privacy right, is there still something wrong?” Medium, April 15. https://medium.com/@TamarSharon/when-google-and-apple-get-privacy-right-is-there-still-something-wrong-a7be4166c295. Accessed 1 July 2020

[CR2] Veale M (2020) “Privacy is not the problem with the Apple-Google contract-tracing app. The Guardian, July 1. https://www.theguardian.com/commentisfree/2020/jul/01/apple-google-contact-tracing-app-tech-giant-digital-rights. Accessed 1 July 2020

[CR3] Winner L (1980). Do artifacts have politics?. Daedalus.

